# Comparisons of clinical characteristics, brain MRI findings, and responses to epidural blood patch between spontaneous intracranial hypotension and post-dural puncture headache: retrospective study

**DOI:** 10.1186/s12883-021-02279-5

**Published:** 2021-06-30

**Authors:** Gha-Hyun Lee, Jiyoung Kim, Hyun-Woo Kim, Jae Wook Cho

**Affiliations:** 1 Department of Neurology, Biomedical Research Institute, Pusan National University Hospital, 179 Gudeok-ro, Seo-gu, Busan, 49241 South Korea; 2 Department of Neurology, Pusan National University Yangsan Hospital, Pusan National University, Yangsan, Republic of Korea

**Keywords:** Spontaneous intracranial hypotension, Post-dural puncture headache, Epidural blood patch, Magnetic resonance imaging

## Abstract

**Background:**

Spontaneous intracranial hypotension and post-dural puncture headache are both caused by **a loss of cerebrospinal fluid** but present with different pathogeneses. We compared these two conditions concerning their clinical characteristics, brain imaging findings, and responses to epidural blood patch treatment.

**Methods:**

We retrospectively reviewed the records of patients with intracranial hypotension admitted to the Neurology ward of the Pusan National University Hospital between January 1, 2011, and December 31, 2019, and collected information regarding age, sex, disease duration, hospital course, headache intensity, time to the appearance of a headache after sitting, associated phenomena (nausea, vomiting, auditory symptoms, dizziness), number of epidural blood patch treatments, and prognosis. The brain MRI signs of intracranial hypotension were recorded, including three qualitative signs (diffuse pachymeningeal enhancement, venous distention of the lateral sinus, subdural fluid collection), and six quantitative signs (pituitary height, suprasellar cistern, prepontine cistern, mamillopontine distance, the midbrain-pons angle, and the angle between the vein of Galen and the straight sinus).

**Results:**

A total of 105 patients (61 spontaneous intracranial hypotension patients and 44 post-dural puncture headache patients) who met the inclusion criteria were reviewed. More patients with spontaneous intracranial hypotension required epidural blood patch treatment than those with post-dural puncture headache (70.5% (43/61) vs. 45.5% (20/44); *p* = 0.01) and the spontaneous intracranial hypotension group included a higher proportion of patients who underwent epidural blood patch treatment more than once (37.7% (23/61) vs. 13.6% (6/44); *p* = 0.007). Brain MRI showed signs of intracranial hypotension in both groups, although the angle between the vein of Galen and the straight sinus was greater in the post-dural puncture headache group (median [95% Confidence Interval]: 85° [68°-79°] vs. 74° [76°-96°], *p* = 0.02).

**Conclusions:**

Patients with spontaneous intracranial hypotension received more epidural blood patch treatments and more often needed multiple epidural blood patch treatments**.** Although both groups showed similar brain MRI findings, the angle between the vein of Galen and the straight sinus differed significantly between the groups.

**Supplementary Information:**

The online version contains supplementary material available at 10.1186/s12883-021-02279-5.

## Background

Spontaneous intracranial hypotension (SIH) and post-dural puncture headache (PDPH) are both caused by **a loss of cerebrospinal fluid** (CSF) but have different pathogeneses. Orthostatic headache is a cardinal symptom that worsens when the patient is upright and improves upon lying down. SIH is caused by CSF leakage from the spinal dura mater without a head trauma history or dural puncture [1]. In contrast, PDPH is caused by injuries or complications of medical procedures such as a lumbar puncture or epidural injection [2].

Brain magnetic resonance imaging (MRI) in patients with SIH can show any combination of pachymeningeal enhancement [3], sinus venous distention [4], subdural fluid collection [5], and pituitary hyperemia [6]. Brain MRI has been used for the diagnosis of SIH because of its ability to identify characteristic abnormalities. However, few reports have described the brain MRI findings for PDPH because this condition is mainly diagnosed based on procedure history and clinical features [7]. Moreover, the imaging findings of SIH and PDPH have rarely been compared.

Although many SIH cases show spontaneous resolution with conservative treatment, autologous epidural blood patch (EBP) injections may be required occasionally. EBP was initially introduced for PDPH treatment but is now also considered as the treatment of choice for SIH. However, the success rate of the first EBP for SIH is only about 30% ~ 60%, and multiple EBPs are frequently required [1, 8, 9]. On the other hand, most cases of PDPH also improve with conservative treatment, and EBP is usually recommended if the symptoms persist for more than ten days [10]. However, in practice, EBP is sometimes performed sooner to facilitate faster resolution of symptoms [11]. Thus, EBP is the currently preferred treatment for PDPH, and the symptoms resolve in about 75% of the cases [12].

SIH and PDPH are both caused by intracranial hypotension, but their pathogenesis and treatment policies differ depending on the disease. To help physicians plan the appropriate treatment approach (conservative treatment or EBP) depending on the disease, we investigated the differences in clinical characteristics, neuroimaging data, and responses to EBP between SIH and PDPH patients.

## Methods

### Demographics and clinical profiles

This retrospective study was conducted using data obtained at our hospital; the institutional review board of Pusan National University Hospital approved this study. Since the study protocol only involved a review of medical records, the necessity for obtaining informed consent was waived. The data in this study were anonymized before its use. All methods were carried out in accordance with relevant guidelines and regulations.

The electronic medical records of consecutive patients with intracranial hypotension who were admitted to the Neurology ward of Pusan National University Hospital between January 1, 2011, and December 31, 2019, were reviewed. Patients were screened based on the diagnosis code at discharge. (including SIH, PDPH, post-lumbar puncture headache, intracranial hypotension, CSF hypovolemia) The inclusion criteria for SIH were as follows [1]; orthostatic headache that worsens within 15 min after sitting or standing and improves < 30 min after recumbent positioning, [2] absence of a procedure or trauma known to be able to cause CSF leakage and [3] the presence of at least one of the following three criteria: low opening CSF pressure (< 60 mm H2O in the sitting position), sustained improvement of symptoms after EBP or evidence of CSF leakage on imaging. The inclusion criteria for PDPH were as follows [1]; orthostatic headache that worsens within 15 min after sitting or standing and improves < 30 min after recumbent positioning, and [2] a history of procedures that induce dural puncture [13–15]. We excluded patients with incomplete medical records. Information regarding age, sex, the interval between symptom onset and diagnosis, hospitalization period, headache intensity (rated on an 11-point [0–10] numeric rating scale), time to the occurrence of a headache after sitting, CSF opening pressure, and associated phenomena (nausea, vomiting, auditory symptoms, and dizziness) was collected.

### Neuroimaging

The routine MRI protocol included native and postcontrast sequences. All images were assessed independently by two authors blinded to the clinical diagnosis. The brain MRI signs of intracranial hypotension were recorded, including three qualitative items (diffuse pachymeningeal enhancement [3], venous distention of the lateral sinus [4], and subdural fluid collection) [5] and six quantitative signs (pituitary height [6], suprasellar cistern, prepontine cistern, mamillopontine distance, the midbrain-pons angle [16], and the angle between the vein of Galen and the straight sinus [vG/SS angle]) [17]. The results recorded by the two authors were aggregated; if the results for qualitative assessments differed between the authors, the existence of the MRI signs was determined using a consensus method. **Another author additionally evaluated the MRI signs, and results were derived based on the majority of opinions (2 of 3) of the binary question.** The average of the results measured by the two authors was used for the quantitative assessments.

### EBP

**Each patient was hospitalized and initially treated with conservative treatments, including bed rest, intravenous fluid infusion, and analgesics. If these conservative managements failed after 3 to 5 days, EBP treatment was initiated.** EBP was performed with a fluoroscopy-guided technique under aseptic conditions by experienced anesthesiologists, who used a 21-gauge epidural needle via a midline approach with the patient in a lateral recumbent position. In patients with SIH, the first EBP was delivered into the thoracolumbar junction. Autologous blood was injected slowly until the onset of radicular pain, headache, nausea, or a maximum injection volume of 30 mL. After the procedure, patients lay in the supine position for at least 2 h. The patients were allowed to stand up and walk the next day. If complete recovery did not occur within three days, an additional EBP was performed. The second EBP was performed into the cervicothoracic junction. If there was no improvement after the second EBP, spine imaging was performed to determine the leakage level, and targeted EBP was performed. In patients with PDPH, the target level of EBP was the most suspected level based on history and medical records. EBP response was defined as complete remission of symptoms within 48 h after the EBP, persisting for at least one month.

### Statistical analysis

Descriptive analysis was performed using frequencies and percentages for categorical variables and median (interquartile range [IQR]) for continuous variables, as well as chi-square, Fisher’s exact, or Mann–Whitney U tests to compare categorical and continuous variables. Differences were considered significant when *p* < 0.05. Interobserver agreement between two authors was calculated using kappa statistics for categorical variables [18] and intraclass correlation coefficient values with a one-way mixed-effects model estimating absolute agreement for continuous variables [19]. All statistical analyses were conducted using MedCalc for Windows, version 19.5.1 (MedCalc Software, Ostend, Belgium).

## Results

A total of 105 patients (45 men and 60 women; median age, 39 years [IQR, 32–52 years]) who met the inclusion criteria were reviewed. The median headache intensity (numeric rating scale) was 7 (IQR, 5.75–8), and the median EBP count was 1.0 (IQR, 0–2). Sixty-one patients (58.1%) had SIH while 44 patients (41.9%) had PDPH.

### SIH patients (supplemental Table 1)

**Fig. 1:**
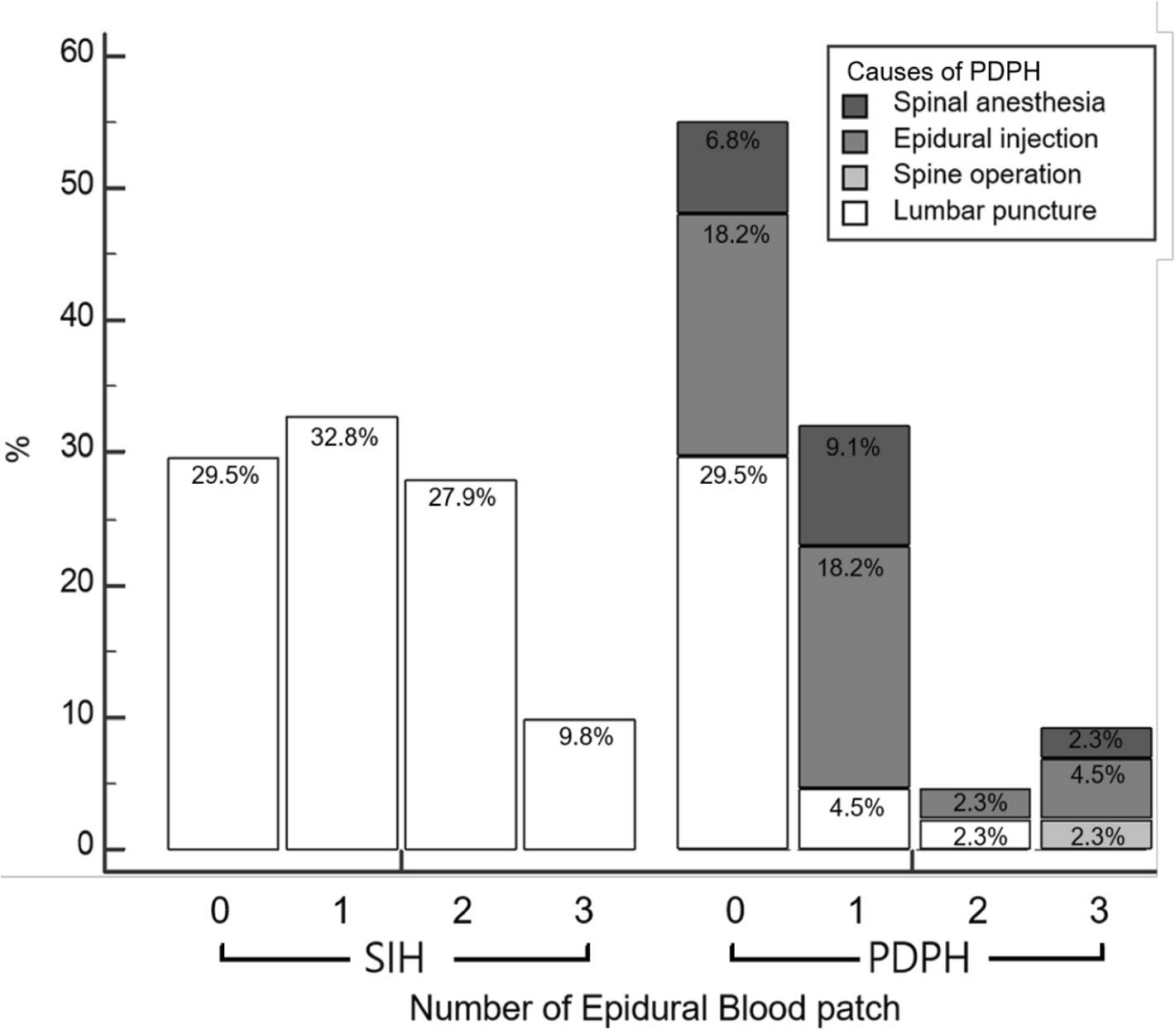
Number of epidural blood patch in patients with SIH and PDPH

Sixty-one patients were diagnosed with SIH, of which 18 (29.5%) improved after conservative treatment. The remaining 43 patients (70.5%) received at least one EBP. The response rate for the first EBP was 46.5% (20/43). Among 23 patients who received the second EBP, 17 (39.5%) responded to the second EBP, and six (14.0%) responded to the third EBP. Two patients (4.7%) responded to the fourth EBP, and one (2.3%) did not respond to the fourth EBP (Table 1, Fig. 1). EBP was performed more frequently in female patients (conservative treatment group: females, 38.9%; EBP group: females, 69.8%; *p* = 0.043). Among the brain MRI findings in SIH patients, the most common abnormalities were diffuse pachymeningeal enhancement (45.3%) and venous distention of the lateral sinus (47.2%). Pachymeningeal enhancement was more frequent in patients who received EBP (conservative treatment group: 25.0%; EBP group: 54.1%; *p* = 0.073); however, the difference was not statistically significant.

**Table 1:** Demographics, clinical features, and EBP response rate in SIH and PDPH patients

Variables	SIH (*n* = 61)	PDPH (*n* = 44)	*p*-value
Age (years)	40 (33–51)	38 (29.5–53)	0.471
Gender			0.615
Male	24 (39.3%)	21 (47.7%)	
Female	37 (40.7%)	23 (52.3%)	
Symptom onset-diagnosis interval (days)	10 (6–15)	4 (3–10.5)	< 0.01
Time to occur a headache after sitting (minutes)	5 (1–10)	8 (4–10)	0.518
Headache intensity (0–10)	7 (5–9)	7 (6–8)	0.651
Associated phenomena			
Nausea	32 (52.5%)	19 (43.2%)	0.317
Vomiting	15 (24.6%)	6 (13.6%)	0.240
Auditory symptoms	12 (19.7%)	3 (6.8%)	0.060
Dizziness	5 (8.2%)	3 (6.8%)	0.661
CSF opening pressure (cmH_2_O)	5.5 (3–8) (*n* = 40)	6.5 (4–9) (*n* = 22)	0.188
Hospitalization period (days)	7 (5–9.25)	6 (4–8.5)	0.087
Treatment			0.015
Conservative management	18 (29.5%)	24 (54.5%)	
EBP once	20 (32.8%)	14 (31.8%)	
EBP twice	17 (27.9%)	2 (4.5%)	
EBP three times	3 (4.9%)	4 (9%)	
EBP four times	3 (4.9%)	0 (0%)	

spontaneous intracranial hypotension (SIH); post-dural puncture headache (PDPH); cerebrospinal fluid (CSF); epidural blood patch (EBP)

Values are presented as the number of patients (%) or median (IQR)

### PDPH patients (supplemental Table 2)

Forty-four patients were diagnosed with PDPH, of which 24 (54.5%) recovered with only conservative treatment. The remaining 20 patients (45.5%) received at least one EBP, and the response rate of the first EBP was 70.0% (14/20). Among the remaining six patients, two (10.0%) responded to the second EBP, and four (20.0%) responded to the third EBP (Table 1, Fig. 1). The most common abnormality in brain MRI of PDPH patients was venous distention of the lateral sinus (39.1%). The interval between symptom onset and diagnosis was longer in patients who received EBP (conservative treatment group: median [IQR], 3 days [2–7 days]; EBP group: 6 days [3–17 days]; *p* = 0.042). Causes of PDPH were significantly different in both groups: lumbar puncture (54.2%) was the most frequent cause in the conservative treatment group, and epidural injection (55.0%) was the most frequent cause in the EBP group (*p* = 0.031). The brain MRI signs of PDPH were not different in the conservative treatment and EBP groups.

### Comparison between SIH and PDPH patients (Tables 1 & 2)

There were no significant differences in age, gender, headache severity, associated symptoms, CSF opening pressure, hospitalization period between SIH and PDPH patients. The interval between symptom onset and diagnosis was longer in SIH patients (median [IQR]: 10 days [6–15 days] vs. 4 days [3–10.5 days]; *p* < 0.001). Auditory symptoms were more frequent in SIH patients (19.7%) than in PDPH patients (6.8%), but the difference was not statistically significant (*p* = 0.06). EBPs were performed more often in the SIH group (43/61, 70.5%) than in the PDPH group (20/44, 45.5%, *p* = 0.01). The number of patients who underwent a second EBP was also higher in the SIH group (23/61, 37.7%) than in the PDPH group (6/44, 13.5%, *p* = 0.007) (Fig. 1). On comparing the brain MRI findings of the SIH and PDPH groups, the vG/SS angle was greater in the PDPH group (median, 85°; IQR, 73°-97°) than in the SIH group (median, 74°; IQR, 50°-84°; *p* = 0.024). Pachymeningeal enhancement (45.3% in SIH, 26.1% in PDPH, *p* = 0.13) and subdural fluid collection (20.8% in SIH, 4.3% in PDPH, *p* = 0.09) were more frequent in SIH patients, but the respective differences were not statistically significant.

**Table 2:** Brain MRI findings in patients with SIH and PDPH

Signs	SIH (*n* = 53)	PDPH (*n* = 23)	p-value	Interobserver Agreement
Qualitative signs				
Pachymeningeal enhancement	24 (45.3%)	6 (26.1%)	0.134	0.692
Venous distention of the lateral sinus	25 (47.2%)	9 (39.1%)	0.618	0.601
Subdural fluid collection	11 (20.8%)	1 (4.3%)	0.093	> 0.99
Angle measurements, midbrain-pons angle, degrees	48 (39–55)	50 (40–59)	0.411	0.775
**≥** 40°	39 (73.6%)	21 (91.3%)	0.132	0.216
< 40°	14 (26.4%)	2 (8.7%)		
vG/SS angle, degrees	74 (60–84)	85 (73–97)	0.020	0.843
Size measurements, mm				
Pituitary height	7.7 (7.0–8.9)	7.6 (6.1–8.6)	0.256	0.768
Suprasellar cistern	4.4 (2.6–5.9)	4.6 (3.9–5.6)	0.483	0.892
Prepontine cistern	4.2 (3.1–5.4)	4.1 (3.5–5.1)	0.955	0.888
Mamillopontine distance	7.1 (5.6–7.8)	6.6 (5.8–8.0)	0.703	0.866

spontaneous intracranial hypotension (SIH); post-dural puncture headache (PDPH); magnetic resonance imaging (MRI); the angle between the vein of Galen and the straight sinus (vG/SS angle)

Values are presented as the number of patients (%) or median (IQR)

## Discussion

This study’s primary finding was that patients with SIH more often received EBPs and more often needed multiple EBPs than patients with PDPH. Most brain MRI findings were similar between SIH and PDPH patients, but the vG/SS angle was significantly different between the two groups.

The SIH and PDPH patients showed no significant differences in clinical characteristics, including hospitalization period. Nausea and vomiting were the most frequent symptoms in patients with SIH and PDPH. The interval between symptom onset and diagnosis was longer in SIH patients, which could be attributed to the fact that PDPH is diagnosed more quickly since the symptoms occur abruptly after the procedure. Although not statistically significant, auditory symptoms were more frequent in SIH patients than in PDPH patients. Because cochleovestibular manifestations occur due to traction or compression of the eighth cranial nerve or a reduction in the pressure of the perilymph, auditory symptoms were observed more often in patients with SIH, which represents chronically advanced intracranial hypotension [1]. Although patients with PDPH received significantly fewer EBPs than those with SIH, they showed no difference in hospitalization period and headache intensity. Our PDPH patients may represent a more severe subgroup of PDPH since we only included admitted PDPH patients, resulting in the lack of a difference in clinical severity between PDPH and SIH.

The characteristic MRI findings for intracranial hypotension include diffuse pachymeningeal enhancement, venous engorgement, and brain sagging [5]. MRI findings are variable, but the association between imaging data and clinical manifestations is not fully understood. There was no statistically significant difference in the incidence of abnormal brain MRI findings in SIH and PDPH patients, but more abnormalities were observed in SIH patients. We speculated that abnormal MRI findings were related to the interval between symptom onset and the MRI scans. Since the MRI findings of intracranial hypotension are compensatory reactions to extradural CSF leakage, patients with a long-standing history of intracranial hypotension are more likely to display atypical clinical and imaging findings [20]. Thus, a variety of disease severity and duration could lead to various combinations of abnormal MRI findings.

The most common brain MRI finding in patients with SIH was diffuse pachymeningeal enhancement, followed by subdural fluid collection. The order of brain MRI abnormalities in our study was similar to that reported previously [21]. In PDPH, brain MRI was thought to show normal findings because the procedures suddenly caused symptoms, but patients with PDPH also showed abnormal brain MRI findings in this study. Venous sinus distension (39.1%) was the most commonly observed abnormal MRI finding in the PDPH group, while the subdural fluid collection was observed in only one patient (4.3%). These findings are presumed to be because venous sinus dilatation occurs early in the intracranial hypotension process, and a chronic change in intracranial hypotension causes subdural fluid collection [22]. The vG/SS angle was significantly greater in the PDPH group than in SIH group. Severe transtentorial brain sagging causes stretching of the vG, which narrows the vG/SS angle. Since brain sagging also reflects a chronic change in intracranial hypotension, the vG/SS angle decreased in SIH, but not in PDPH patients [17].

EBP is the treatment of choice for patients who have not responded to conservative management for intracranial hypotension [1]. The efficacy of the first EBP for SIH is about 30% ~ 60%, and multiple EBPs are often required [1, 8, 9]. On the other hand, the efficacy of EBP in PDPH is much better, and the first EBP has a success rate of approximately 70 to 90% [23]. Even with accidental dural tears due to epidural catheterizations, the efficacy of response to EBP is better than that in SIH. This could be because, in PDPH, EBP is typically targeted right at or very close to the leakage site [24]. In contrast, the leakage site in SIH is mostly present at the levels above the lumbar spine where the EBPs are placed. Moreover, the dural defect in SIH is not a simple hole but occurs through three main mechanisms: meningeal diverticular lesions, ventral dural tears, and CSF-venous fistula [25]. Although targeted EBP in SIH may be more successful than blinded EBP [9], there is controversy over whether targeted or blind EBP is better for SIH treatment [26]. EBP in the lumbar area is safer than that in other areas and blinded EBP can be performed without other invasive or expensive tests to confirm the leakage site. **It is argued that the goal of blind lumbar EBP is not to seal and repair the CSF leakage site, but to reverse the CSF pressure-venous pressure gradient within the spinal epidural space** [27]**. Ohtonari et al. reported that a large-volume EBP, using an intravenous catheter at a single lumbar entry point, provided complete CSF leak control in 14 SIH and 1 traumatic CSF leak patients with suspected multiple CSF leakage sites** [28]**.** Even in our study, blinded EBP was safe and effective. The response rate of conservative management and up to two blinded EBP treatments was 90% in all SIH patients. The number of patients who underwent EBP three or more times was similar in the SIH (9.8%) and PDPH (9.1%) groups.

In the SIH group, EBP was performed more frequently in women, consistent with the results published in another study [29]. **Under the influence of estrogen, swelling of the cerebral blood vessels in CSF hypovolemia is likely to be more pronounced in young women, leading to more severe headaches** [30]. **Moreover, there are reports that pain sensitivity may be higher in women** [31]**, so EBP is more likely to be required by women**. Although not statistically significant due to the small number of patients, SIH patients who underwent EBP showed more pachymeningeal enhancement and venous distension of the lateral sinus and quantitative signs that suggest brain sagging more frequently than patients received conservative management (supplemental Table 1).

Among PDPH patients, the EBP group showed a significantly longer interval between symptom onset and diagnosis than the conservative management group (supplemental Table 2). EBPs were likely performed earlier without conservative management in patients who already had symptoms for an extended period. The number of EBPs significantly differed according to the cause of PDPH. Among patients who developed PDPH by lumbar puncture, symptoms improved by only conservative management without EBP in most cases (13 out of 16), maybe because there was only a stab injury without significant damage to the dura mater. Among patients with PDPH caused by procedures other than a diagnostic lumbar puncture, such as epidural injection, EBP was frequently performed in 59.3% (16 of 27 patients); since epidural injection induces dural tears and spinal anesthesia involves an injection of drugs into the spinal cavity, these procedures are more invasive than a diagnostic lumbar puncture.

The present study had several limitations. First, the number of patients included in this study was small. If the number of patients was larger, more clinical characteristics and MR findings might have shown significant differences between SIH and PDPH patients. Second, there were fewer brain MRI studies in PDPH patients. Among SIH patients, 86.9% (53 of 61) underwent brain MRI, whereas, among PDPH patients, only 52.3% (23 of 44) underwent brain MRI. Third, this was a retrospective study, and a more extensive prospective study is needed to confirm these results.

## Conclusions

Our study showed no difference in clinical characteristics between the SIH and PDPH groups. The vG/SS angle in the brain MRI, which reflects brain sagging, was significantly narrower in the SIH group than in the PDPH group. The success rate of EBP in SIH patients was lower than that of PDPH, and more patients with SIH received EBP treatments and needed multiple EBP treatments than patients with PDPH. Notably, in PDPH patients, the cause of PDPH was important. Most patients with PDPH due to lumbar puncture improved symptoms with conservative management without EBP. SIH and PDPH can be treated without significant complications if they are managed with appropriate investigation and treatment. It is a clinical decision to provide EBP to patients who have failed conservative treatment; therefore, information regarding clinical characteristics and MRI findings is necessary for treatment decisions.

## Supplementary Information

**Additional file 1.**

## Data Availability

The datasets used and analyzed during the study are available from the corresponding author on reasonable request.

## Abbreviations

SIH: spontaneous intracranial hypotension
PDPH: post-dural puncture headache
CSF: cerebrospinal fluid
MRI: magnetic resonance imaging
EBP: epidural blood patch
vG/SS angle: the angle between the vein of Galen and the straight sinus
