# Supplementary material for: Comparisons of clinical characteristics, brain MRI findings, and responses to epidural blood patch between spontaneous intracranial hypotension and post-dural puncture headache: retrospective study
Source: BMC Neurol. 2021 Jun 30;21:253. doi: 10.1186/s12883-021-02279-5 (PMC8243531; doi:10.1186/s12883-021-02279-5)
Supplement: Supplementary file 1 — Additional file 1. [file 12883_2021_2279_MOESM1_ESM.docx]

**Title**

Comparisons of clinical characteristics, brain MRI findings, and responses to epidural blood patch between spontaneous intracranial hypotension and post-dural puncture headache: Retrospective study

**Authors**

Gha-Hyun Lee, MD, Jiyoung Kim, MD, PhD, Hyun-Woo Kim, MD^1^, Jae Wook Cho, MD, PhD^1^

Department of Neurology, Biomedical Research Institute, Pusan National University Hospital, Busan, Korea

^1^Department of Neurology, Pusan National University Yangsan Hospital, Pusan National University, Yangsan, Republic of Korea.

Table 1. Characteristics of SIH patients.

| Variables | Conservative treatment (n=18) | EBP (n=43) | p |
| --- | --- | --- | --- |
| Age (years) | 39 (33-46) | 43 (33-52) | 0.447 |
| Gender |  |  |  |
| Male | 11 (61.1%) | 13 (30.2%) | 0.043 |
| Female | 7 (38.9%) | 30 (69.8%) |  |
| Symptom onset-diagnosis interval (days) | 8.5 (6-14) | 10 (7-14) | 0.757 |
| Time to occur a headache after sitting (minutes) | 5 (1-10) | 8 (4-10) | 0.980 |
| Headache intensity (0-10) | 5 (5-9) | 5 (6-9) | 0.533 |
| Associated phenomena |  |  |  |
| Nausea | 7 (38.9%) | 25 (58.1%) | 0.261 |
| Vomiting | 5 (27.8%) | 10 (23.3%) | 0.750 |
| Auditory symptoms | 2 (11.1%) | 10 (23.3%) | 0.481 |
| Dizziness | 1 (5.6%) | 4 (9.3%) | >0.999 |
| SDH | 1 (5.6%) | 2 (4.7%) | >0.999 |
| CSF opening pressure (cmH_2_O) | 7 (5-9) (n=12) | 5 (3-7) (n=28) | 0.191 |
| Hospitalization period (days) | 5 (4-8) | 8 (6-10) | 0.011 |
| Brain MRI | n=16 | n=37 |  |
| Qualitative signs (n, %) |  |  |  |
| Pachymeningeal enhancement | 4 (25.0%) | 20 (54.1%) | 0.073 |
| Venous distention of the lateral sinus | 5 (31.2%) | 20 (54.1%) | 0.147 |
| Subdural fluid collection | 3 (18.8%) | 8 (21.6%) | >0.999 |
| Angle measurements |  |  |  |
| midbrain-pons angle, median (IQR), degrees  $\boldsymbol{\geq}$ 40° (n, %)  < 40° (n, %) | 51 (44-66)  14 (87.5%)  2 (12.5%) | 46 (37-54)  25 (67.6%)  12 (32.4%) | 0.214  0.201 |
| vG/SS angle, median (IQR), degrees | 77 (67-86) | 73 (58-83) | 0.399 |
| Size measurements, median (IQR), mm |  |  |  |
| Pituitary height | 7.13 (6.7-8.5) | 8.3 (7.2-9.0) | 0.261 |
| Suprasellar cistern | 4.9 (3.9-5.7) | 4.0 (2.4-6.2) | 0.314 |
| Prepontine cistern | 5.0 (3.6-5.7) | 3.7 (2.8-5.4) | 0.146 |
| Mamillopontine distance | 7.3 (6.5-8.6) | 6.5 (5.5-7.5) | 0.108 |

spontaneous intracranial hypotension (SIH); cerebrospinal fluid (CSF); magnetic resonance imaging (MRI); epidural blood patch (EBP); the angle between the vein of Galen and the straight sinus (vG/SS angle)

Values are presented as the number of patients (%) or median (IQR, Interquartile range)

Table 2. Characteristics of PDPH patients.

| Variables | Conservative treatment (n=24) | EBP (n=20) | p |
| --- | --- | --- | --- |
| Age (years) | 33 (29-48) | 43 (34-57) | 0.092 |
| Gender |  |  |  |
| Male | 10 (41.7%) | 11 (55.0%) | 0.545 |
| Female | 14 (58.3%) | 9 (45.0%) |  |
| Symptom onset-diagnosis interval (days) | 3 (2-7) | 6 (3-17) | 0.042 |
| Time to occur a headache after sitting (minutes) | 5 (2-10) | 10 (5-10) | 0.477 |
| Causes |  |  | 0.031 |
| Lumbar puncture | 13 (54.2%) | 3 (15.0%) |  |
| Epidural injection | 8 (33.3%) | 11 (55.0%) |  |
| Spinal anesthesia | 3 (12.5%) | 4 (20.0%) |  |
| Spine operation | 0 (0%) | 1 (5.0%) |  |
| Headache intensity (0-10) | 7 (7-8) | 7 (5-8) | 0.661 |
| Associated phenomena |  |  |  |
| Nausea | 10 (41.7%) | 9 (45.0%) | >0.999 |
| Vomiting | 4 (16.7%) | 2 (10.0%) | 0.673 |
| Auditory symptoms | 0 (0%) | 3 (15.0%) | 0.086 |
| Dizziness | 2 (8.3%) | 1 (5.0%) | >0.999 |
| SDH | 0 (0%) | 0 (0%) | >0.999 |
| CSF opening pressure (cmH_2_O) | 8 (6-9) (n=9) | 6 (4-10) (n=13) | >0.999 |
| Hospitalization period (days) | 6 (4-7) | 7 (5-10) | 0.168 |
| Brain MRI | n=10 | n=13 |  |
| Qualitative signs (n, %) |  |  |  |
| Pachymeningeal enhancement | 1 (10.0%) | 5 (38.5%) | 0.179 |
| Venous distention of the lateral sinus | 5 (50.0%) | 4 (30.8%) | 0.417 |
| Subdural fluid collection | 0 (0%) | 1 (7.7%) | >0.999 |
| Angle measurements |  |  |  |
| midbrain-pons angle, median (IQR), degrees  $\boldsymbol{\geq}$ 40° (n, %)  < 40° (n, %) | 55 (41-63)  10 (100%)  0 (0%) | 50 (40-56)  11 (84.6%)  2 (15.4%) | 0.401  0.204 |
| vG/SS angle, median (IQR), degrees | 79 (70-90) | 89 (86-105) | 0.072 |
| Size measurements, median (IQR), mm |  |  |  |
| Pituitary height | 7.7 (6.5-8.8) | 7.0 (5.5-8.5) | 0.556 |
| Suprasellar cistern | 4.5 (3.7-5.7) | 4.6 (4.4-5.6) | 0.733 |
| Prepontine cistern | 4.0 (3.1-4.2) | 4.4 (3.7-5.5) | 0.239 |
| Mamillopontine distance | 5.9 (5.6-8.1) | 7.3 (6.2-8.0) | 0.438 |

post-dural puncture headache (PDPH); cerebrospinal fluid (CSF); magnetic resonance imaging (MRI); epidural blood patch (EBP); the angle between the vein of Galen and the straight sinus (vG/SS angle)

Values are presented as the number of patients (%) or median (IQR, Interquartile range)
